# Aspirin for Primary Prevention of Cardiovascular Events: Meta-Analysis of Randomized Controlled Trials and Subgroup Analysis by Sex and Diabetes Status

**DOI:** 10.1371/journal.pone.0090286

**Published:** 2014-10-31

**Authors:** Manling Xie, Zhilei Shan, Yan Zhang, Sijing Chen, Wei Yang, Wei Bao, Ying Rong, Xuefeng Yu, Frank B. Hu, Liegang Liu

**Affiliations:** 1 Department of Nutrition and Food Hygiene, Hubei Key Laboratory of Food Nutrition and Safety, School of Public Health, Tongji Medical College, Huazhong University of Science & Technology, Wuhan, China; 2 Ministry of Education Key Laboratory of Environment and Health, School of Public Health, Tongji Medical College, Huazhong University of Science & Technology, Wuhan, China; 3 Division of Endocrinology, Department of Internal Medicine, Tongji Hospital, Tongji Medical College, Huazhong University of Science and Technology, Wuhan, China; 4 Department of Nutrition and Epidemiology, Harvard School of Public Health, Boston, Massachusetts, United States of America; Indiana University School of Medicine, United States of America

## Abstract

**Objective:**

To evaluate the benefits and harms of aspirin for the primary prevention of CVD and determine whether the effects vary by sex and diabetes status.

**Methods:**

We searched Medline, Embase, and Cochrane databases for randomized controlled trials comparing the effects of aspirin with placebo or control in people with no pre-existing CVD. Two investigators independently extracted data and assessed the study quality. Analyses were performed using Stata version 12.

**Results:**

Fourteen trials (107,686 participants) were eligible. Aspirin was associated with reductions in major cardiovascular events (risk ratio, 0.90; 95% confidence interval, 0.85–0.95), myocardial infarction (0.86; 0.75–0.93), ischemic stroke (0.86; 0.75–0.98) and all-cause mortality (0.94; 0.89–0.99). There were also increases in hemorrhagic stroke (1.34; 1.01–1.79) and major bleeding (1.55; 1.35–1.78) with aspirin. The number needed to treat to prevent 1 major cardiovascular event over a mean follow-up of 6.8 years was 284. By comparison, the numbers needed to harm to cause 1 major bleeding is 299. In subgroup analyses, pooled results demonstrated a reduction in myocardial infarction among men (0.71; 0.59–0.85) and ischemic stroke among women (0.77; 0.63–0.93). Aspirin use was associated with a reduction (0.65; 0.51–0.82) in myocardial infarction among diabetic men. In meta-regression analyses, the results suggested that aspirin therapy might be associated with a decrease in stroke among diabetic women and a decrease in MI among diabetic men and risk reductions achieved with low doses (75 mg/day) were as large as those obtained with higher doses (650 mg/day).

**Conclusions:**

The use of low-dose aspirin was beneficial for primary prevention of CVD and the decision regarding an aspirin regimen should be made on an individual patient basis. The effects of aspirin therapy varied by sex and diabetes status. A clear benefit of aspirin in the primary prevention of CVD in people with diabetes needs more trials.

## Introduction

The burden of cardiovascular disease (CVD) is substantial. The most recent (2013) statistics on heart disease and stroke from the American Heart Association (AHA) estimate that the annual direct and indirect cost of CVD and stroke in the United States alone are $523 billion [1]. From 2000 to 2010, the total number of inpatient cardiovascular operations and procedures increased 28%, from 5,939,000 to 7,588,000. By 2030, 40.8% of the US population is projected to have some form of CVD, and the annual cost will increase to $1.13 trillion [1]. These strong upward trends underline the importance of primary prevention for those who are already at high risk of CVD.

The use of low-dose aspirin for primary prevention of CVD is recommended by many key guidelines [2], [3]. However, a recent published study and a review [4], [5] stated that the benefit of aspirin for the primary prevention of cardiovascular events was relatively small for individuals regardless of diabetic status and could easily be offset by the risk of hemorrhage. These studies challenge current recommendations, which are based on outcomes from several meta-analyses [6]–[10], prompting re-evaluation of the efficacy of aspirin. An important sex-specific meta-analysis showed that the effects of aspirin varied by sex [6]. However, it was conducted in 2006 and included only six primary prevention trials. In addition, the results were not confirmed in the Antithrombotic Trialists' Collaboration meta-analysis and a recent publication which did not find significant sex different in treatment effect [7], [11]. Several guidelines [12], [13] recommend aspirin for the primary prevention of cardiovascular events in patients with diabetes at risk of CVD, but others [2], [14] do not. This conflict reflects the lack of definitive evidence. Existing recommendations are primarily based on extrapolations from indirect evidence, given the absence of statistically significant results in published meta-analyses in diabetics [15]–[23].

Therefore, we performed a new meta-analysis to re-assess the effects of aspirin for primary prevention of CVD and to investigate whether the effects vary by sex and diabetes status. Compared to the previous sex-specific meta-analyses, we enrolled almost twice that of previously published data. Given the limited power to detect interactions, even in a meta-analysis combining the results from several studies [24], we used multiple statistical methods to examine the diabetes-aspirin interaction and sex-aspirin interaction and their consistent results strengthen our conclusions.

## Methods

For this meta-analysis, we used methods and definitions from previous meta-analyses [6] and performed our meta-analysis in line with approach recommended by the PRISMA statement [25]. Full study protocol is provided as Text S1.

### Data Sources and Searches

Randomized controlled trials (RCTs) comparing the effect of aspirin with placebo or control in people without pre-existing CVD on outcomes of interest were eligible for inclusion. We identified trials by searching Medline, Embase, and Central (the Cochrane Central Register of Controlled Trials) from inception to December 2012. Reference lists from previously published relevant systematic reviews were also screened for additional studies [6], [8], [15]. The search strategies are as follows: First we searched terms “aspirin*” [MeSH] and term “primary prevention”. Then the Boolean term “AND” was used to combine these two terms. Highly sensitive filters were used to limit results to randomized controlled trials and human studies. We searched only studies published in English. A similar search strategy was used for Embase and Central.

### Study Selection

Two authors independently reviewed search results by title and abstract, then full text to identify eligible trials. Selection criteria included: (1) Prospective, randomized, controlled, open, or blinded trials. (2) Participants without clinical CVD (e.g., established or symptomatic) were randomly assigned to aspirin (any dose) versus placebo or control group for the primary prevention of CVD. (3) Trials carried out on a background of anticoagulation were eligible. (4) Follow-up had to exceed 90 days, because such short follow-up would not permit detection of cardiovascular outcomes related to aspirin treatment for primary prevention.

### Outcomes

The outcomes of interest for both aspirin and control groups included major cardiovascular events (MCE, defined as death from cardiovascular causes, nonfatal myocardial infarction, and nonfatal stroke); myocardial infarction (MI, fatal and nonfatal); stroke (fatal and nonfatal; ischemic and hemorrhagic); ischemic stroke; cardiovascular mortality; total mortality (death from any cause); hemorrhagic stroke and major bleeding. Definitions for major bleeding varied across studies. However, participant-level data was unavailable to allow reclassification according to standard criteria [26]–[28]. Among all bleeding events, the gastrointestinal hemorrhage is one of the most common and serious complications of long-term aspirin use.

### Data Extraction and Quality Assessment

Two investigators independently extracted data and evaluated the methodological quality using criteria previously published [29]. An arbitrator settled discrepancies by discussion in accordance with our selection criteria. We collected some basic information on the studies and outcomes of interest listed above. Data were collected from the original articles, previously published meta-analyses, and through contact with study authors.

### Data Synthesis and Analysis

Analyses were performed using Stata version 12 (Stata Corp). Heterogeneity was assessed by Cochran's Q-test and the *I^2^* statistic [30]. A *P* value less than 0.10 indicated significant heterogeneity. For the *I^2^* metric, we defined low, moderate, and high *I^2^* values as 25%, 50%, and 75%, respectively [31]. We estimated the results with pooled relative risks (RR) and 95% confidence intervals (CI) using a Mantel-Haenszel fixed-effect model when the heterogeneity was negligible or moderate and a DerSimonian and Laird random-effects model when heterogeneity was significant [32]. All analyses were based on the intension-to-treat principle. A 2-tailed *P*-value 0.05 was considered statistically significant.

To explore potential sources of heterogeneity, we collected sufficient information to conduct particular subgroup analyses to determine the sex-aspirin interaction and diabetes-aspirin interaction. Because there is limited power to detect interactions, even in a meta-analysis combining the results from several studies, and it is not sufficient to conclude that the relative risks from the subgroups significantly different from each other when the two estimates and *P* values seem very different [24]. Thus, we implemented two methods to determine whether a difference exists in subgroup analysis. First, we estimated the pooled ratio of RRs comparing the aspirin effect in patients with and without diabetes and in patients with different genders across trials. Second, we used the method of Altman and Bland to compare the pooled RR and its 95% CI across subgroups [24]. In addition, we calculated numbers needed to treat (NNT) and numbers needed to harm (NNH) to examine the risk vs. benefit of aspirin therapy for some endpoints [33]. Values of NNT and NNH provided herein represent the number of persons that need to be treated with aspirin for 6.8 years (the overall mean follow-up time in our study) to avert or incur, respectively, 1 event.

We also performed meta-regression analyses to appraise the impact of gender and the daily dose of aspirin on outcomes [34]. Publication bias was assessed by the funnel plot and the Begg's and Egger's tests. We performed a sensitivity analysis to examine the robustness of the results, systematically removing one study from the analysis and recalculating the results.

## Results

### Description of Trials

**Figure 1:**
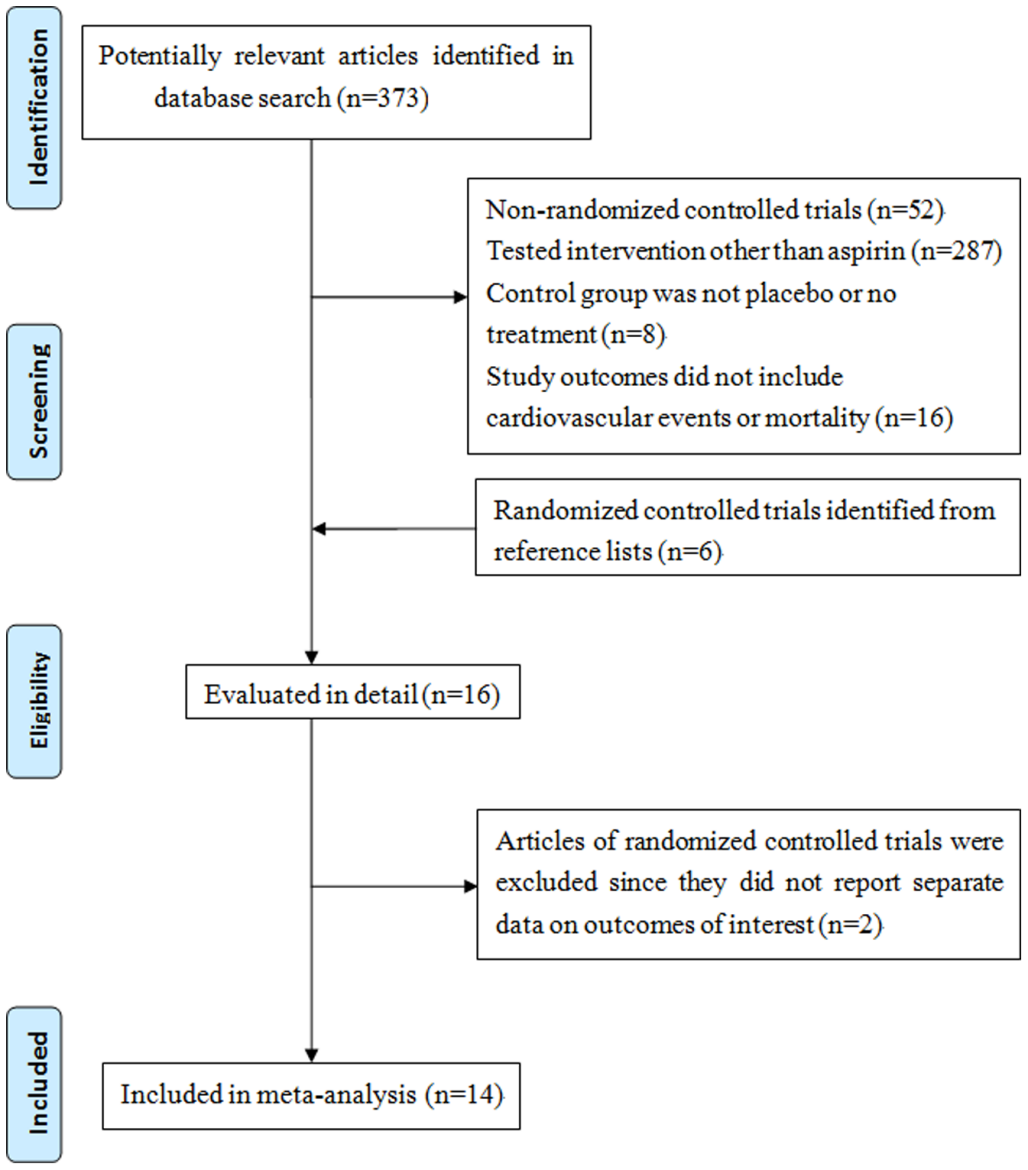
Flow chart of articles selection for this systematic review and meta-analysis.

Details of the included studies appear in Table 1. Table S1 outlines the baseline characteristics and the interventions of the participants. We identified fourteen [35]–[48] prospective randomized controlled trials comprised 107, 686 participants for inclusion from 373 potentially eligible studies (Figure 1). A total of 734,170 person-years of exposure were recorded: 372757 in the aspirin group and 361413 in the placebo or control group. Specifically, three trials included apparently healthy health care professionals [35], [36], [43]. Only one of the 14 studies included a small proportion (<10%) of participants with pre-existing established cardiovascular events [37]. In addition, few studies have populations with high prevalence of CVD risk factors, e.g., hypertension [40], polycythemia vera [42], and peripheral arterial disease [44].

**Table 1:** Design of trials included in the meta-analysis.

Studies	Year of publication	Country	No. of participants	No. of Aspirin group	Patients population	Mean years of follow-up	Aspirin dose	Primary outcome measure
BDT [35]	1988	UK	5139	3429	Healthy male doctors	5.6	500 mg daily or 300 mg if requested	Cardiovascular mortality, nonfatal MI, stroke
PHS [36]	1989	USA	22071	11037	Healthy male doctors	5	325 mg every other day	Cardiovascular mortality
ETDRS [37]	1992	Mixed	3711	1856	Men and women with diabetes	5	650 mg daily	All cause mortality
ACBS [38]	1995	Unclear	372	188	Individuals with asymptomatic carotid stenosis	2.4	325 mg daily	Clinical event in the composite end point
TPT [39]	1998	UK	5085	2545	Men at high risk of CVD	6.7*	75 mg daily	All ischemic heart disease (coronary death and fatal and nonfatal MI)
HOT [40]	1998	Mixed	18790	9399	Men and women at high risk of hypertension	3.8	75 mg daily	Cardiovascular mortality, nonfatal MI, stroke
PPP [41]	2001	Italy	4495	2226	Men and women>1 cardiovascular risk factor	3.7	100 mg daily	Cardiovascular mortality, MI, stroke
ECLAP [42]	2004	Unclear	518	253	Patients with polycythemia vera	3	100 mg daily	Two composite end point
WHS [43]	2005	USA	39876	19934	Healthy women	10.1	100 mg every other day	Cardiovascular mortality, nonfatal MI, stroke
CLIPS [44]	2007	European	366	185	Patients with peripheral arterial disease	2	100 mg daily	MCEs
APLASA [45]	2007	Mixed	98	48	Asymptomatic, persistently antiphospholipid	2.3	81 mg daily	Incident acute thrombosis (arterial or venous) confirmed by appropriate imaging studies
POPADAD [46]	2008	UK	1276	638	Men and women with diabetes and ABI ≤0.96	6.7	100 mg daily	Cardiovascular mortality, nonfatal MI, stroke,critical limb ischemia
JPAD [47]	2008	Japan	2539	1262	Men and women with diabetes	4.4*	81 or 100 mg daily	All ischemic heart disease, stroke and peripheral artery disease.
AAA [48]	2010	UK	3350	1675	Men and women in general population with ABI≤0.95	8.2	100 mg daily	Cardiovascular mortality, MI, stroke and revascularization

*Median year follow-up.

PHS = Physicians Health Study. BDT = British Doctor's Trial. TPT = Thrombosis Prevention Trial. HOT = Hypertension Optimal Treatment trial. PPP = Primary Prevention Project.WHS = Women's Health Study. POPADAD = Prevention of Progression of Arterial Disease and Diabetes trial. JPAD = Japanese primary Prevention of Atherosclerosis with Aspirin for Diabetes trial. AAA = Aspirin for Asymptomatic Atherosclerosis trial. ETDRS = the Early Treatment Diabetic Retinopathy Study. APLASA = Antiphospholipid Antibody Acetyl-salicylic Acid study. ECLAP = European Collaboration on Low-Dose Aspirin in Polycythemia Vera study. CLIPS = Critical Leg Ischaemia Prevention Study. ACBS = Asymptomatic Cervical Bruit Study.

MCEs = major cardiovascular events; MI = myocardial infarction.

### Risk of Bias in Individual Trials

The risk of bias in trials is presented in Table S2. Randomization was stated in all studies, but the allocation concealment was adequately described in only eight studies and unclear in the remainder. Two trials were open-labeled [41], [47], and placebo tables were not used in the control group in one trial [35]. Outcome assessment was not blinded in one trial [35] and unclear in two [42], [45]. The description of incomplete outcome data was not adequate in two trials [36], [43]. Three studies had a vitamin component [41], [43], [44], one had a beta carotene component [36], one had a anti-oxidant component [46], and one had a warfarin component [39].

### Clinical Outcomes

#### Efficacy Data: Major Cardiovascular Events

**Figure 2:**
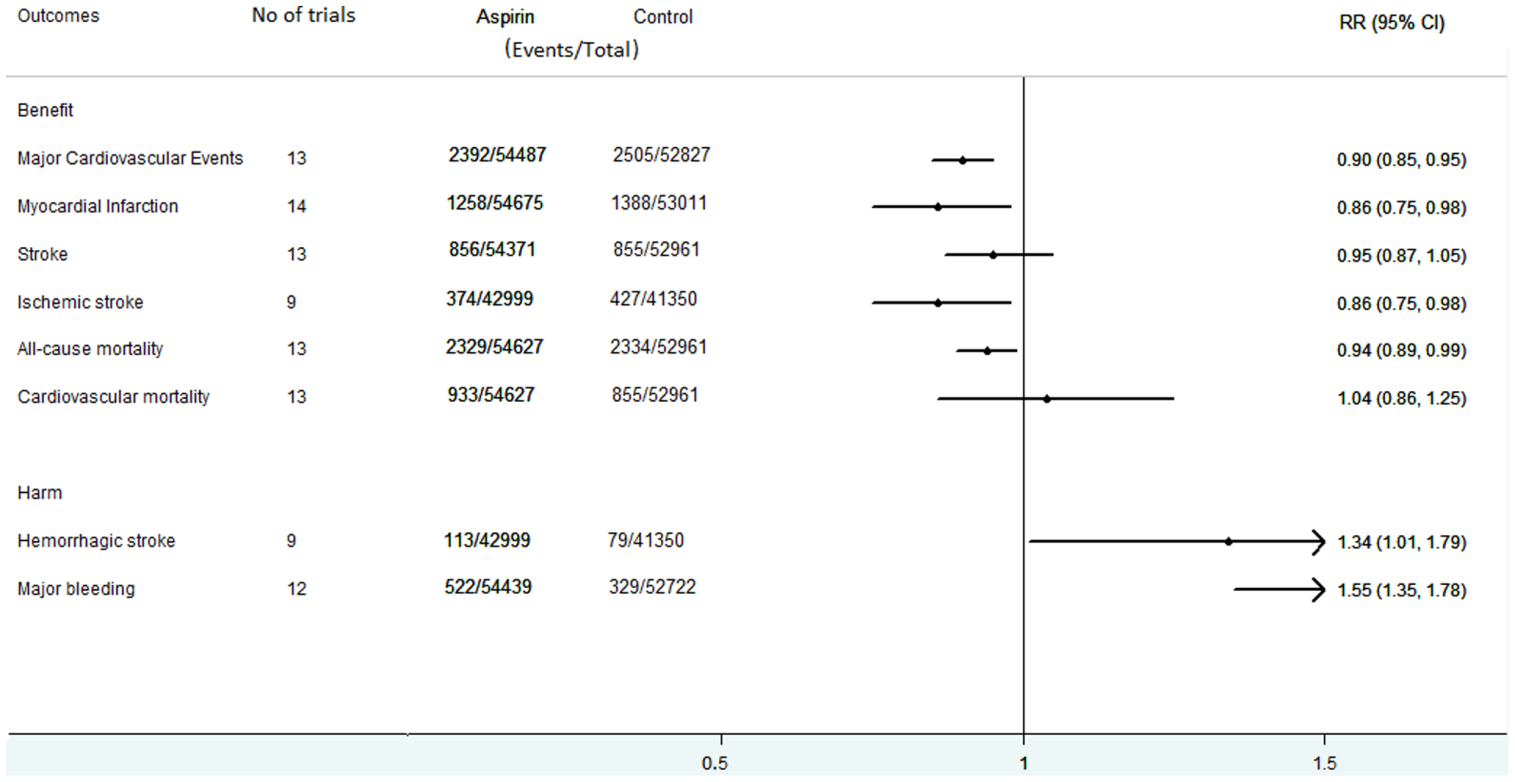
Effect of aspirin therapy versus placebo or control for primary prevention of CVD. MCE = major cardiovascular events; MI = myocardial infarction; CVD = cardiovascular disease.

Aspirin use was associated with a 10% reduction in MCEs (No. of events/No. of totals, 2392/54487 vs 2505/52827; RR, 0.90; 95% CI, 0.85 to 0.95; *P*<0.01; Figure 2, Figure S4-A in File S1). The NNT to avoid 1 MCE over 6.8 years was 284. There was no significant heterogeneity among the studies in this analysis (Q = 14.17, *P* = 0.29; *I^2^* = 15.3%).

#### Myocardial Infarction

There was also a 14% reduction in the risk of MI with aspirin (1258/54675 vs 1388/53011; RR, 0.86; 95% CI, 0.75 to 0.98; *P* = 0.02; Figure 2, Figure S4-B in File S1). The NNT to avoid 1 MI over 6.8 years was 315. However, heterogeneity was significant (Q = 28.17, *P* = 0.01; *I^2^* = 53.9%).

#### Stroke

There was no reduction in the risk of overall stroke (856/54371 vs 855/52961; RR, 0.95; 95% CI, 0.87 to 1.05; *P* = 0.34; Figure 2, Figure S4-C in File S1) and no significant heterogeneity (Q = 14.33, *P* = 0.28; *I^2^* = 16.3%). When we examined stroke subtypes (ischemic and hemorrhagic) from the available data, we found a 14% reduction (374/42999 vs 427/41350; RR, 0.86; 95% CI, 0.75 to 0.98; *P* = 0.03; Figure 2, Figure S4-D in File S1) in the risk of ischemic stroke without significant heterogeneity (Q = 9.60, *P* = 0.30; *I^2^* = 16.6%). The NNT to avoid 1 l ischemic stroke over 6.8 years was 614.

#### All-Cause and Cardiovascular Mortality

Pooled results demonstrated a 6% reduction in the risk of all-cause mortality (2329/54627 vs 2334/52961; RR, 0.94; 95% CI, 0.89 to 0.99; *P* = 0.03; Figure 2, Figure S4-E in File S1). The NNT to avoid 1all-cause mortality over 6.8 years was 697. The heterogeneity was not significant (Q = 5.87, *P* = 0.92; *I^2^* = 0%). However, there was no reduction in cardiovascular mortality (933/54627 vs 855/52961; RR, 1.04; 95% CI, 0.86 to 1.25; *P* = 0.69; Figure 2, Figure S4-F in File S1) and the heterogeneity was significant (Q = 32.68, *P*<0.01; *I^2^* = 63.3%).

#### Safety Data: Hemorrhagic stroke

Aspirin was associated with a 34% increase in hemorrhagic stroke (113/42999 vs 79/41350; RR, 1.34; 95% CI, 1.01 to 1.79; *P* = 0.05; Figure 2, Figure S4-G in File S1). The NNH to cause 1 hemorrhagic stroke over 6.8 years was 1394. Heterogeneity was not significant (Q = 3.89, *P* = 0.87; *I^2^* = 0%).

#### Major bleeding

Pooled results demonstrated a 55% increase in the risk of major bleeding (522/54439 vs 329/52722; RR, 1.55; 95% CI, 1.35 to 1.78; *P*<0.01; Figure 2, Figure S4-H in File S1). The NNT to cause 1 major bleeding over 6.8 years was 299. In aggregate, heterogeneity was moderate in this analysis (Q = 17.47, *P* = 0.10; *I^2^* = 37.0%).

### Subgroup Analysis

#### The effects of aspirin by gender

Details of the included studies in the subgroup analyses by sex appear in Table S3.

For the endpoint of MCE, aspirin was associated with a 12% reduction (879/28575 vs 998/28643; RR, 0.88; 95% CI, 0.81 to 0.96; *P* = 0.01) among women, and a 12% reduction (1368/25426 vs 1394/23688; RR, 0.88; 95% CI, 0.82 to 0.95; *P*<0.01) among men, without significant heterogeneity (Table 2).

**Table 2:** Outcomes of subgroup analyses by sex and diabetes status.

Outcomes	Aspirin Control Aspirin Control				Rate ratio (95% CI)		*I^2^* (%)		*P* value*
	(No. of events/No. of totals)				(Aspirin vs Control)				
	**Male**		**Female**		**Male**	**Female**	**Male**	**Female**	
MCEs	1368/25426	1394/23688	879/28575	998/28643	0.88(0.82–0.95)	0.88(0.81–0.96)	8.6	0	0.5
MI	616/23953	760/22257	316/26473	334/26484	0.71(0.59–0.85)	0.94(0.80–1.09)	61.1	10	0.02
Stroke	406/23953	320/22257	319/26217	374/26484	1.13(0.98–1.31)	0.86(0.74–1.00)	0	0	0.01
Ischemic stroke	141/17960	129/16274	176/21211	230/21248	1.02(0.80–1.30)	0.77(0.63–0.93)	23.5	0	0.08
Hemorrhagic stroke	50/17960	25/16247	51/21211	43/21248	1.69(1.05–2.72)	1.19(0.79–1.77)	0	25.4	0.27
Cardiovascular mortality	539/24239	480/22534	276/26825	303/26845	0.97(0.86–1.10)	0.90(0.77–1.06)	3.9	12.4	0.47
All-cause mortality	1046/23953	981/22257	836/26473	903/26484	0.93(0.85–1.01)	0.92(0.84–1.01)	0	65.4	0.56
Major bleeding	195/22922	102/21227	183/25648	118/25694	1.79(1.41–2.27)	1.55(1.23–1.96)	0	36.6	0.4
	**Diabetes**		**Non-diabetes**		**Diabetes**	**Non-diabetes**	**Diabetes**	**Non-diabetes**	
MCEs	615/5663	698/5601	1285/35626	1268/34021	0.92(0.83–1.01)	0.91(0.84–0.98)	0	0	0.87
MI	406/5840	457/5788	517/42142	631/42250	0.85(0.66–1.10)	0.84(0.67–1.04)	54.1	66.9	0.94
Stroke	221/5938	236/5859	620/47762	595/46429	0.92(0.77–1.10)	0.98(0.87–1.09)	27.2	0	0.56
Cardiovascular mortality	313/5027	345/5031	150/11984	162/12031	0.91(0.97–1.05)	0.82(0.45–1.49)	45.5	75.8	0.74
All-cause mortality	533/5027	561/5031	447/11984	500/12031	0.95(0.85–1.06)	0.90(0.79–1.02)	0	26.4	0.53
	**Diabetes rate <50%**		**Diabetes rate>50%**		**Diabetes rate <50%**	**Diabetes rate>50%**	**Diabetes rate <50%**	**Diabetes rate >50%**	
MCEs	1876/50546	1973/48876	516/3941	568/3951	0.90(0.84–0.95)	0.91(0.82–1.01)	5.6	46.5	0.86
MI	913/50734	998/49060	345/3941	390/3951	0.85(0.72–0.99)	0.88(0.65–1.20)	56.2	57	0.84
Stroke	695/50430	688/49010	161/3941	167/3951	0.95(0.86–1.06)	0.97(0.78–1.19)	20.4	29.8	0.87
Ischemic stroke	347/41099	400/39435	27/1900	27/1915	0.85(0.73–0.98)	1.01(0.60–1.71)	30.8	0	0.54
Hemorrhagic stroke	106/41099	73/39435	7/1900	6/1915	1.35(1.01–1.82)	1.18(0.40–3.49)	0	0	0.81
Cardiovascular mortality	648/50686	540/49010	285/3941	315/3951	1.06(0.85–1.32)	0.95(0.58–1.55)	65.9	57.8	0.69
All-cause mortality	1854/50686	1825/49010	475/3941	509/3951	0.94(0.88–1.00)	0.93(0.83–1.05)	0	0	0.87
Major bleeding	441/50498	257/48771	81/3941	72/3951	1.67(1.43–1.94)	1.12(0.82–1.54)	0	49.1	0.03

*For interaction.

Pooled results demonstrated a 29% reduction (616/23953 vs 760/22257; RR, 0.71; 95% CI, 0.59 to 0.85; *P*<0.01; Q = 12.86, *P* = 0.03; *I^2^* = 61.1%) in the risk of MI among men and a 23% reduction (176/21211 vs 230/21248; RR, 0.77; 95% CI, 0.63 to 0.93; *P* = 0.01; Q = 0.05, *P* = 0.82; *I^2^* = 0%) in the risk of ischemic stroke among women (Table 2).

For hemorrhagic stroke with aspirin, pooled results demonstrated no significant increase (51/21211 vs 43/21248; RR, 1.19; 95% CI, 0.79 to 1.77; *P* = 0.41) among women but a 69% increase (50/17960 vs 25/16247; RR, 1.69; 95% CI, 1.05 to 2.72; *P* = 0.03) among men (Table 2). There was no significant heterogeneity among the studies in this analysis.

Aspirin use was also associated with a significant risk of major bleeding irrespective of sex. Pooled results demonstrated a 55% increase (183/25648 vs 118/25694; RR, 1.55; 95%CI, 1.23 to 1.96; *P*<0.01; Q = 3.15, *P* = 0.21; *I^2^* = 36.6%) among women and a 79% increase (195/22922 vs 102/21227; RR, 1.79; 95%CI, 1.41 to 2.27; *P*<0.01; Q = 2.27, *P* = 0.69; *I^2^* = 0%) among men (Table 2).

When we used the method of Altman and Bland to compare the pooled RR and its 95% CI of MI (*P* = 0.02) and stroke (*P* = 0.01), the results also provide strong support for gender difference in the reduction of MI and stroke.

#### The effects of aspirin by diabetes status

Details of the included studies in the subgroup analyses by diabetes status appear in Table S4-A and Table S4-B.

The estimate stratified by diabetes status was significant only for the outcome of MCEs. Pooled results demonstrated a 9% reduction (1285/35626 vs 1268/34021; RR, 0.91, 95% CI, 0.84 to 0.98, *P* = 0.01) among nondiabetic patients but no significant reduction among diabetic patients (Table 2). Given that the small number of the diabetic patients, we stratified the trials by the percentage of diabetic patients (<50% vs>50%). For trials with percentage of diabetic patients <50% and>50%, the RRs of MI were 0.85 (95% CI 0.72 to 0.99; *P* = 0.04; Q = 20.57, *P* = 0.02; *I^2^* = 56.2%) and 0.88 (95% CI,0.65 to 1.20; *P* = 0.42; Q = 6.97, *P* = 0.07; *I^2^* = 57.0%) respectively, and the RRs of major bleeding were 1.67 (95% CI, 1.43 to 1.94; *P*<0.01; Q = 5.78, *P* = 0.57; *I^2^* = 0%) and 1.12 (95% CI, 0.82 to 1.54; *P* = 0.46; Q = 5.89, *P* = 0.12; *I^2^* = 49.1%), respectively (Table 2). When we used the method of Altman and Bland to compare the pooled RRs and their 95% CIs of major bleeding (*P* = 0.03), the result demonstrated different treatment effects in trials according to the percentage of diabetic patients.

Among diabetic patients, we also conducted stratified analysis by sex. Pooled results demonstrated a 35% reduction in MI among men (RR, 0.65; 95% CI, 0.51 to 0.82; *P*<0.01; Q = 3.21, *P* = 0.20; *I^2^* = 37.6%), but the results were not significant in women (RR, 0.90; 95% CI, 0.71 to 1.14; *P* = 0.37; Q = 4.01, *P* = 0.14; *I^2^* = 50.1%). When we used the method of Altman and Bland to compare the pooled RR and its 95% CI (*P* = 0.06), the results demonstrated that difference in the reduction of MI was not significant. The data was insufficient to estimate other endpoints in diabetic patients.

### Meta-regression

**Figure 3:**
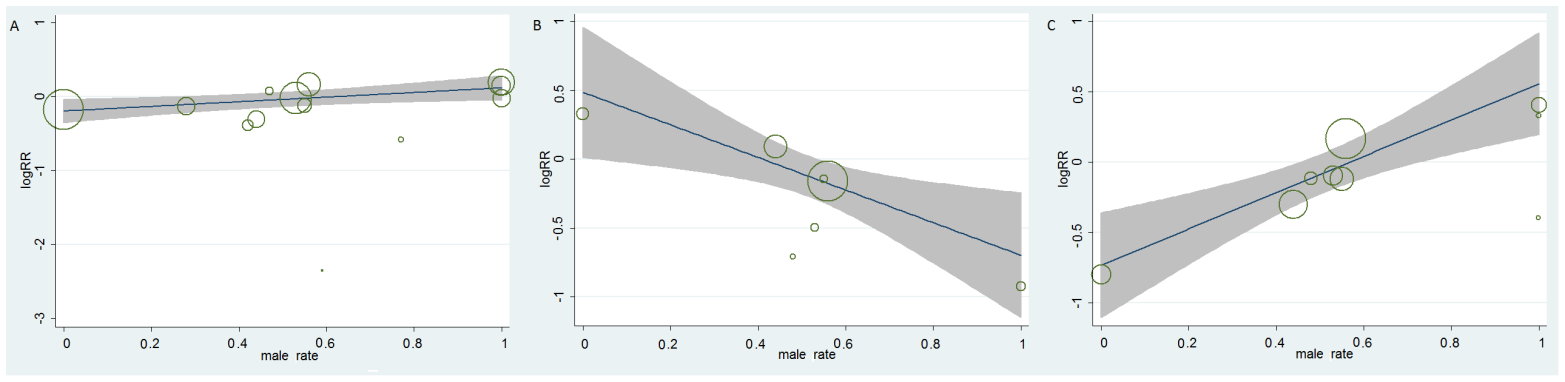
Meta-regression between male percentage and the effects of aspirin on risk of MI or stroke. (A) Log relative risk of stroke in relation to male percentage in all people. (B) Log relative risk of MI in relation to male percentage in diabetic patients. (C) Log relative risk of stroke in relation to male percentage in diabetic patients. The gray bonds in each figure are confidence interval. The size of the bubble represents the value of the weight. MI = myocardial infarction.

First we performed meta-regression in all populations to appraise the impact of the percentage of males on the incidence of endpoints. There was a statistically significant relationship between percentage of males and the effect of aspirin on stroke (*P* = 0.04) (Figure 3A), which supported the conclusions from subgroup analysis. With respect to the negative results among patients with diabetes, we then performed meta-regression in diabetic patients. The result demonstrated a significant association between percentage of males and the effects of aspirin on MI (*P* = 0.03) (Figure 3B) and stroke (*P* = 0.02) (Figure 3C), suggesting that aspirin therapy might be associated with a decrease in stroke among diabetic women and a decrease in MI among diabetic men.

We also performed meta-regression to test for a linear relation of the positive effects (reduction in MCE, MI, ischemic stroke) or complications (major bleeding and hemorrhagic stroke) with daily dose of aspirin. The results suggested that risk reductions achieved with low doses (75 mg/day) were as large as those obtained with higher doses (650 mg/day), and the risk for bleeding did not increase with dose (Figure S1).

### Sensitivity Analysis

The heterogeneity was significant for the outcome of MI (Q = 28.17, *P* = 0.01; *I^2^* = 53.9%), which may be explained by the fact that WHS enrolled only women (weight 13.61%) and the PHS enrolled only men (weight 16.86%). The RR was 0.84 and 0.91 when these two studies were respectively removed from the model (Figure S2). After excluding these two studies from our analyses, heterogeneity between trials substantially decreased (921/23704 vs 956/22035; RR, 0.88; 95%CI, 0.81 to 0.96; *P*<0.01, Q = 11.14, *P* = 0.43; *I^2^* = 1.3%).

### Publication Bias

We used a comprehensive literature search strategy to minimize the risk of publication bias. Results of Begg's and Egger's tests for asymmetry were not statistically significant (Figure S3).

## Discussion

In a comparison of aspirin with placebo or control for the prevention of CVD, we found significant benefits of 10, 14, and 14% risk reduction for the outcomes of MCEs, MI, and ischemic stroke respectively in the overall population. Meanwhile, there were also clear harms of 34% relative increase in hemorrhagic stroke and 55% relative increase in major bleeding events. Our subgroup and meta-regression analyses indicated that the effects of aspirin therapy varied by sex and diabetes status. Aspirin use was associated with a significant reduction in the risk of cardiovascular events in both sexes but different specific types of benefits: a reduction in MI among men and a reduction in ischemic stroke among women. Aspirin had no significant effect on CVD in the overall diabetic population, but was associated with a reduction in MI among men with diabetes.

Although the results indicate a significant increase in bleeding complications, it is not sensible to conclude that the benefit of aspirin is offset by the risk of bleeding. First, we should estimate not only the incidence of benefits and harms, but also take into account the consequences of both harms and benefits on quality of life [49]. Setting aside the potentially fatal MI or stroke, it is clear that a non-fatal stroke or MI is more likely to result in long-term disability than a non-fatal gastrointestinal or other extracranial bleed. Although serious intracranial and extracranial bleed may also cause serious results, our results suggest that the benefit of reducing risk of ischemic stroke outweigh the harm hemorrhagic stroke. In addition, there are several methods to mitigate these adverse effects, for example, clinicians can remind those patients who decide to begin or continue an aspirin regiment for primary prevention of CVD, of the early recognition of the signs and symptoms as well as the risk factors of gastrointestinal bleeding. These risk factors include age, gender, upper gastrointestinal tract pain, gastrointestinal ulcers, NSAID use, uncontrolled hypertension, concomitant use of anticoagulants, and family history of gastrointestinal ulcers and so on [50].

Second, some of the previous published trials were criticized that few subjects exceeded the threshold for aspirin prophylaxis recommended by the American Heart Association [51]. In addition, evidence shows that>60% of aspirin users were above 60 years of age, 4–6% had a recent history of peptic ulcers, and over 13% used other non-steroidal antiinflammatory drugs [52]. It is obvious that the gastrointestinal harms would outweigh the cardiovascular benefits in certain groups whose gastrointestinal risk is high but cardiovascular risk is low. Thus, some of the previous published trials may overestimate the harm effect of the aspirin.

Third, the Antithrombotic Trialists' Collaborative, an individual-level meta-analysis of RCTs, indicated that the absolute benefits of aspirin were on a small order of magnitude in primary prevention and the effects of aspirin do not significantly depend on smoking history, blood pressure, total cholesterol, body-mass index, history of diabetes, or predicted risk of coronary heart disease [7]. However, the small number and rare events in these particular subgroups are not sufficient for precise estimate, and thus this paper provides insufficient evidence to answer the question of which particular category of individuals derive the most benefit from aspirin therapy. More highly powered analyses for specific populations are expected based on two major ongoing trials: the Aspirin to Reduce Risk of Initial Vascular Events (ARRIVE) Study (http://www.arrive-study.com/EN/study.cfm) and Aspirin in Reducing Events in the Elderly (ASPREE) [53].

The results of our subgroup analysis consistent with prior studies indicate that there is no significant benefit of aspirin therapy among patients with diabetes, but this may be due to inadequate power because the point estimate was similar to that among nondiabetics but with a wider confidence interval. It is well established that diabetes mellitus is associated with an increased risk of CVD [54]. Among diabetes patients, the coagulation system is altered, because plasma levels of procoagulant factors are increased while fibrinolytic capacity is decreased [55].

The mechanisms of the antithrombotic effects of low-does aspirin involve two aspects: cyclooxygenase (COX)-dependent actions and COX-independent actions [56]. Low-dose aspirin is considered to induce a permanent inactivation of COX-1 which results in the inhibition of platelet aggregation [57], [58]. In many people, generation of new platelets and recovery of COX-dependent platelet aggregation can reverse to a certain degree this effect within 24 hours after administration of aspirin [59]. Thus successive and low-dose daily administration of aspirin is essential to maintain inactivation of platelet COX-1. However, patients with type 2 diabetes have been demonstrated to be characterized by a large inter-individual variability in the recovery of COX-1 activity and enhanced platelet turnover rate which represents an important determinant of the extent and duration of platelet inhibition on repeated dosing with low-dose aspirin [60]–[63]. Thus it is possible that the current use of a once-a-day and low-dose regimen may not be sufficient to induce clinical benefits among diabetic patients [64]–[67]. More studies are needed to demonstrate whether a higher frequency of aspirin administration and possibly a higher daily dosage can optimize treatment with aspirin in diabetic patients. In addition, considerable efforts are needed to illuminate the relation between decreased responsiveness to aspirin and the COX-independent antithrombotic effects [56].

Platelet dysfunction, increased platelet aggregation and aspirin insensitivity were more common in patients with type 2 diabetes compared to nondiabetic people [68] [62]. In addition, insulin resistance and hyperglycaemia are reported to contribute to these alterations [55]. Among our eligible 14 studies, 6 were published before 2000. The management and treatments of diabetes have been improved over the decades. Thus, the treatments of diabetes are much different between the old studies and recent studies. These differences of the treatments may have impact on the effect of aspirin in diabetes subgroup analysis.

Our meta-regression analyses indicate that aspirin therapy may have different effects between the sexes in diabetic patients. Although there is no evidence that the pharmacodynamics of platelet inhibition by aspirin is any different in women than in men, the overall risk of CVD for people with diabetes is reported to be increased two-to threefold in men, and three-to fivefold in women [23]. More highly powered subgroup analyses for specific populations are awaited based on two major ongoing trials: A Study of Cardiovascular Events in Diabetes (ASCEND, International Standard Randomised Controlled Trial Number ISRCTN60635500, http://www.ctsu.ox.ac.uk/ascend/) and the Aspirin and Simvastatin Combination for Cardiovascular Events Prevention Trial in Diabetes (ACCEPT-D, Current Controlled Trials ISRCTN48110081) [69], which enrolled more than 15,000 diabetic patients without prior cardiovascular events to assess the effect of aspirin in the prevention of cardiovascular events. These trials may provide sufficient data to identify patients who derive the most benefit from aspirin therapy.

In sex subgroup analysis, our results are consistent with the previous sex-specific meta-analysis [6], but our findings conflicted with a recent publication which did not find any significant sex different in treatment effect [7], [11]. There are several potential explanations for the different effects between sexes.

First, the different epidemiologic characteristics of cardiovascular disease between men and women may contribute to the different benefits. After age 40 years, men have a 49% lifetime risk for a coronary heart disease event, while women have a 32% risk. Men have a higher risk for MI, while the lifetime risk for ischemic stroke is greater in women than men from age 55 to 75 (17–18% in women and 13–14% in men) [2], and the risk of gastrointestinal bleeding is approximately twice as high in men than women [50].

Second, although some evidence indicates that there is no difference in pharmacodynamics of platelet inhibition by aspirin between the sexes and the ‘aspirin resistance’ may not exist [70]–[72], there is still insufficient evidence to support these conclusions. In fact, few randomized trials have measured ‘aspirin resistance’ directly, whether gender plays an important part in ‘aspirin resistance’ remains a question for future research [73]–[76].

The range of the dosage varies from 75 to 650 mg/day in eligible trials. Our analysis suggests that risk reductions achieved with low doses (75 mg/day) were similar to those obtained with higher doses (650 mg/day). In fact, it is reported that the successive daily administration of 30 mg of aspirin is sufficient to result in virtually inactivation of COX-1 [77]. However, there is no trial using this dosage. It is reported that apart from the inhibition of platelet aggregation, the impairment of cytoprotection in the gastrointestinal mucosa which is clearly dose-dependent also increases risk of upper gastrointestinal bleeding associated with aspirin therapy [72]. Thus, it is very likely that lower dosage of aspirin would decrease the bleeding complications. There is a need for additional placebo-controlled trials to demonstrate whether lower dosage of aspirin should be recommended for primary prevention of CVD in the future. However, because of the varied definitions of major bleeding among the studies, the result of our meta-regression does not indicate a clear does-effect relation which conflicts with previous published studies [78], [79].

We acknowledge several limitations of our studies. First, we observed moderate heterogeneity among trials for some outcomes of interest. However, we have no access to patient-level data and our author response rate was relatively low, which may have led to limited statistical power. Second, the data were insufficient to report separate outcomes for type 1 and type 2 diabetes and different sexes in diabetic patients. Finally, the data on bleeding in our analyses were not sufficient to estimate whether changes in the dose of aspirin might reduce the risk of hemorrhage and whether further attempts at dosage reduction may compromise therapeutic efficacy.

In conclusion, our results demonstrate a significant net benefit to risk of aspirin for the primary prevention of CVD, and the decision regarding an aspirin regimen should be made on an individual patient basis, after careful evaluation of the trade-off between benefits and harms by the physician and patient. The effects of aspirin therapy vary by sex. Additional evidence is necessary before we make specific recommendations for aspirin use according to diabetes status.

## Supporting Information

Figure S1 **Meta-regression between the effects or complications of aspirin and daily dose of aspirin.** (A) Log relative risk of MCEs in relation to daily dose of aspirin. (B) Log relative risk of MI in relation to daily dose of aspirin. (C) Log relative risk of ischemic stroke in relation to daily dose of aspirin. (D) Log relative risk of major bleeding in relation to daily dose of aspirin. (E) Log relative risk of hemorrhagic stroke in relation to daily dose of aspirin.
(TIF)

Figure S2 **The result of sensitivity analysis for the outcome of myocardial infarction.**
(TIF)

Figure S3 **Funnel plots of effect estimates for various clinical outcomes.**
(TIF)

Table S1 **Patient characteristics included in the meta-analysis.**
(DOCX)

Table S2 **Quality assessment of eligible trials comparing aspirin with placebo or control.**
(DOCX)

Table S3 **Details of the included studies in the subgroup analyses by sex.**
(DOCX)

Table S4 A. Details of the included studies in the subgroup analyses by diabetes status. B. Details of the included studies in the subgroup analyses by diabetes rate.
(DOCX)

Checklist S1 PRISMA 2009 Checklist.
(DOC)

Text S1 **Study protocol.**
(DOC)

File S1 **Forest plot of effect of aspirin.** Figure S4 in File S1. Figure S4-A Effect of aspirin on the prevention of MCEs. Figure S4-B MI. Figure S4-C Stroke. Figure S4-D Ischemic stroke. Figure S4-E All-cause mortality. Figure S4-F Cardiovascular mortality. Figure S4-G Hemorrhagic stroke. Figure S4-H Major bleeding.
(RAR)
