# Supplementary figures and images for: Aspirin for Primary Prevention of Cardiovascular Events: Meta-Analysis of Randomized Controlled Trials and Subgroup Analysis by Sex and Diabetes Status
Source: PLoS One. 2014 Oct 31;9(10):e90286. doi: 10.1371/journal.pone.0090286 (PMC4215843; doi:10.1371/journal.pone.0090286)

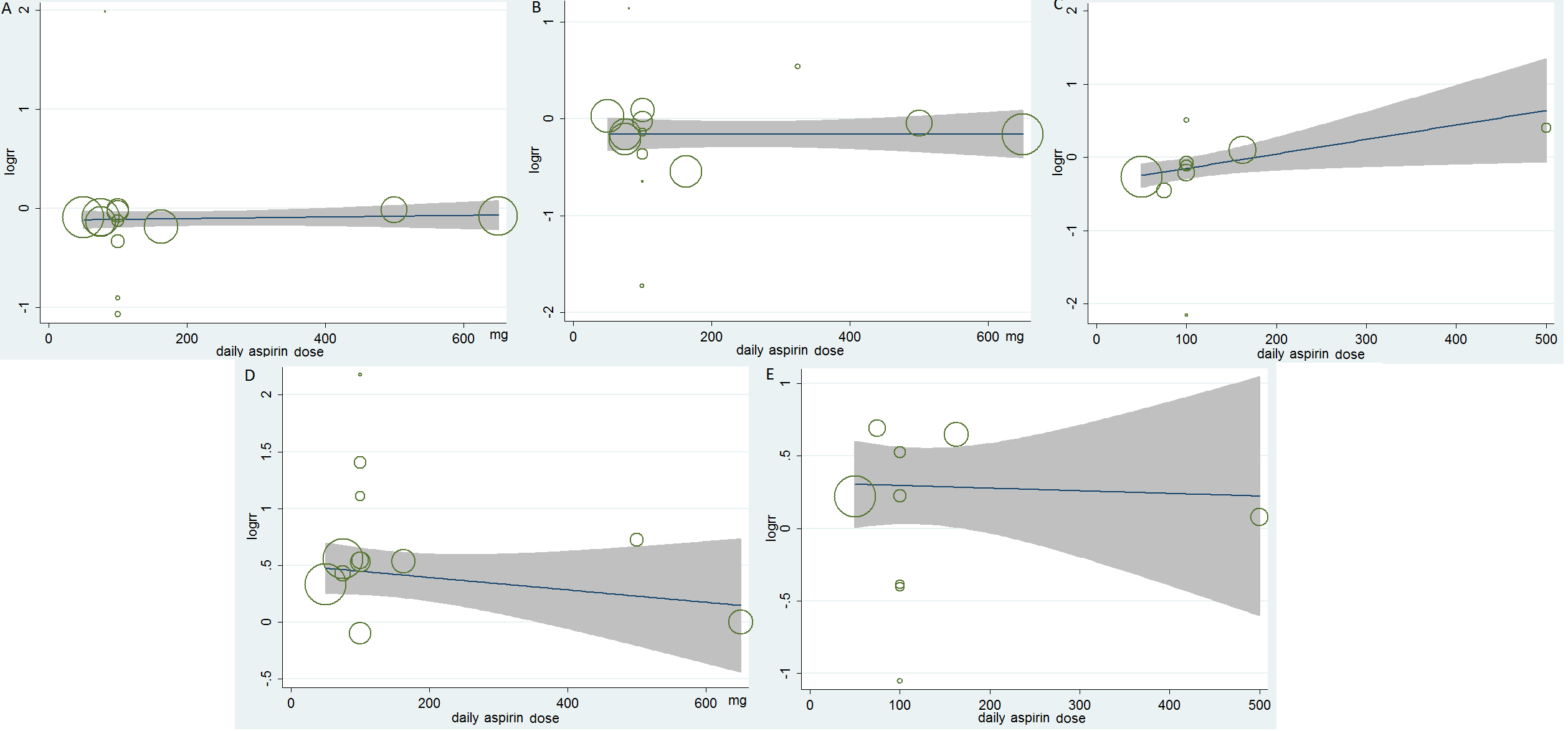

Supplement: Figure S1 — Meta-regression between the effects or complications of aspirin and daily dose of aspirin. (A) Log relative risk of MCEs in relation to daily dose of aspirin. (B) Log relative risk of MI in relation to daily dose of aspirin. (C) Log relative risk of ischemic stroke in relation to daily dose of aspirin. (D) Log relative risk of major bleeding in relation to daily dose of aspirin. (E) Log relative risk of hemorrhagic stroke in relation to daily dose of aspirin. (TIF) [file pone.0090286.s001.tif]

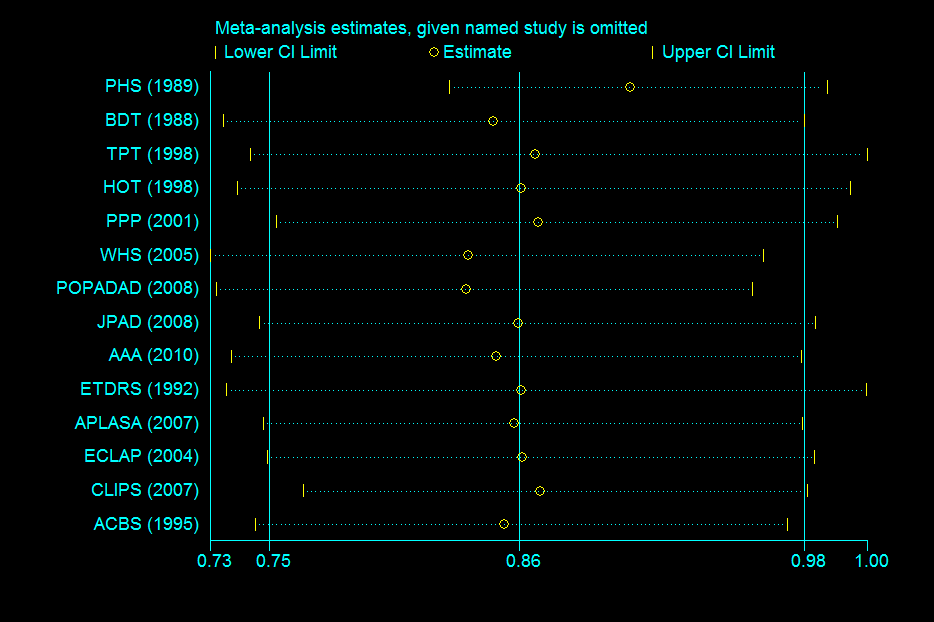

Supplement: Figure S2 — The result of sensitivity analysis for the outcome of myocardial infarction. (TIF) [file pone.0090286.s002.tif]

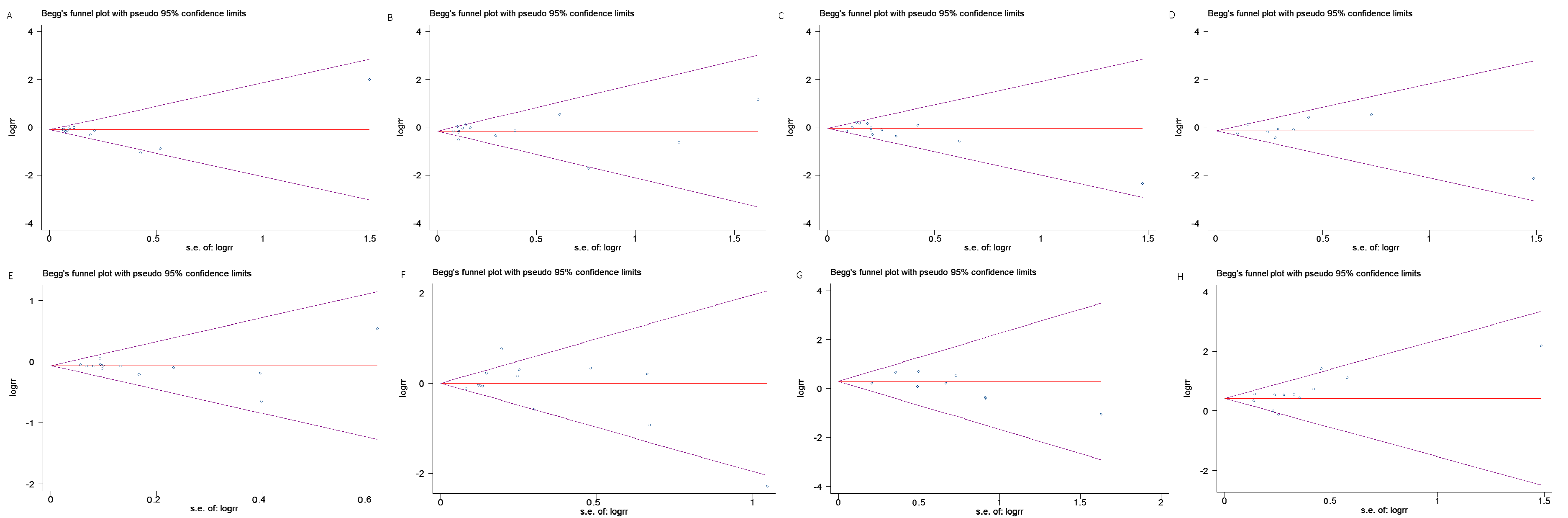

Supplement: Figure S3 — Funnel plots of effect estimates for various clinical outcomes. (TIF) [file pone.0090286.s003.tif]
