# Supplementary material for: Aspirin for Primary Prevention of Cardiovascular Events: Meta-Analysis of Randomized Controlled Trials and Subgroup Analysis by Sex and Diabetes Status
Source: PLoS One. 2014 Oct 31;9(10):e90286. doi: 10.1371/journal.pone.0090286 (PMC4215843; doi:10.1371/journal.pone.0090286)
Supplement: Table S1 — Patient characteristics included in the meta-analysis. (DOCX) [file pone.0090286.s004.docx]

**Table S1. Patient characteristics included in the meta-analysis.**

| **Studies** | **Male rate** | **No. of diabetic patients** | **Diabetes rate** | **Age** | **BMI(kg/m²）** | **Smoker (%)** | **Hypertension (%)** | **Total cholesterol (mg/dL)** |
| --- | --- | --- | --- | --- | --- | --- | --- | --- |
| PHS | 1 | 533 | 0.024 | 53 | 24.9 | 11 | 9 | 212 |
| BDT | 1 | 101 | 0.02 | 50-78 | Unclear | 13 | 10 | Unclear |
| TPT | 1 | 68 | 0.013 | 57.5 | 27.4 | 41 | 26 | 247 |
| HOT | 0.53 | 1501 | 0.08 | 61.5 | 28.4 | 16 | 100 | 236 |
| PPP | 0.42 | 1031 | 0.299 | 64 | 27.6 | 15 | 68 | 236 |
| WHS | 0 | 1037 | 0.026 | 55 | 26 | 13 | 26 | Unclear |
| POPADAD | 0.44 | 1276 | 1 | 60 | 24 | 31 | Unclear | 213 |
| JPAD | 0.55 | 2539 | 1 | 65 | 29 | 21 | Unclear | 201 |
| AAA | 0.28 | 88 | 0.026 | 62 | Unclear | 65 | Unclear | 240 |
| ETDRS | 0.56 | 3711 | 1 | 18-70 | Unclear | 44 | Unclear | Unclear |
| APLASA | 0.1 | 6 | 0.061 | ≥18 | Unclear | 14 | 22 | Unclear |
| ECLAP | 0.59 | 25 | 0.048 | 61 | 25.6 | 16 | 35 | Unclear |
| CLIPS | 0.77 | 277 | 0.76 | 66 | Unclear | Unclear | 61 | Unclear |
| ACBS | 0.47 | 88 | 0.24 | 66 | Unclear | 37 | 51 | Unclear |

PHS = Physicians Health Study. BDT = British Doctor’s Trial. TPT = Thrombosis Prevention Trial. HOT = Hypertension Optimal Treatment trial. PPP = Primary Prevention Project.WHS = Women’s Health Study. POPADAD = Prevention of Progression of Arterial Disease and Diabetes trial. JPAD = Japanese Primary Prevention of Atherosclerosis With Aspirin for Diabetes Trial. AAA = Aspirin for Asymptomatic Atherosclerosis trial. ETDRS = the Early Treatment Diabetic Retinopathy Study. APLASA = Antiphospholipid Antibody Acetyl-salicylic Acid study. ECLAP = European Collaboration on Low-Dose Aspirin in Polycythemia Vera study. CLIPS = Critical Leg Ischaemia Prevention Study. ACBS = Asymptomatic Cervical Bruit Study.
