# Supplementary material for: Aspirin for Primary Prevention of Cardiovascular Events: Meta-Analysis of Randomized Controlled Trials and Subgroup Analysis by Sex and Diabetes Status
Source: PLoS One. 2014 Oct 31;9(10):e90286. doi: 10.1371/journal.pone.0090286 (PMC4215843; doi:10.1371/journal.pone.0090286)
Supplement: Table S2 — Quality assessment of eligible trials comparing aspirin with placebo or control. (DOCX) [file pone.0090286.s005.docx]

**Table S2. Quality assessment of eligible trials comparing aspirin with placebo or control**

| Studies | Random sequence generation | Allocation concealment | Blinding of participants or investigators | Blinding of outcome assessment | Incomplete outcome data | Selective outcome reporting | Other threats to validity |
| --- | --- | --- | --- | --- | --- | --- | --- |
| PHS | Yes | Unclear | Yes | Yes | No | Yes | No^*^ |
| BDT | Yes | Yes | No | No | Yes | Yes | Yes |
| TPT | Yes | Unclear | Yes | Yes | Yes | Yes | No^†^ |
| HOT | Yes | Yes | Yes | Yes | Yes | Yes | No^**^ |
| PPP | Yes | Yes | No | Yes | Yes | Yes | No^*^ |
| WHS | Yes | Unclear | Yes | Yes | No | Yes | No^*^ |
| POPADAD | Yes | Yes | Yes | Yes | Yes | Yes | No^‡^ |
| JPAD | Yes | Yes | No | Yes | Yes | Yes | Yes |
| AAA | Yes | Yes | Yes | Yes | Yes | Yes | Yes |
| ETDRS | Yes | Unclear | Yes | Yes | Yes | Yes | Yes |
| APLASA | Yes | Yes | Yes | Unclear | Yes | Yes | Yes |
| ECLAP | Yes | Unclear | Yes | Unclear | Yes | Yes | No^§^ |
| CLIPS | Yes | Yes | Yes | Yes | Yes | Yes | No^*^ |
| ACBS | Yes | Unclear | Yes | Yes | Yes | Yes | Yes |

PHS = Physicians Health Study. BDT = British Doctor’s Trial. TPT = Thrombosis Prevention Trial. HOT = Hypertension Optimal Treatment trial. PPP = Primary Prevention Project.WHS = Women’s Health Study. POPADAD = Prevention of Progression of Arterial Disease and Diabetes trial. JPAD = Japanese Primary Prevention of Atherosclerosis with Aspirin for Diabetes Trial. AAA = Aspirin for Asymptomatic Atherosclerosis trial. ETDRS = the Early Treatment Diabetic Retinopathy Study. APLASA = Antiphospholipid Antibody Acetyl-salicylic Acid study. ECLAP = European Collaboration on Low-Dose Aspirin in Polycythemia Vera study. CLIPS = Critical Leg Ischaemia Prevention Study. ACBS = Asymptomatic Cervical Bruit Study.

* comparator of vitamin E

† comparator of warfarin

** comparator of antihypertensive agent

‡comparator of anti-oxidant table

§other recommended treatments
