# Supplementary material for: Aspirin for Primary Prevention of Cardiovascular Events: Meta-Analysis of Randomized Controlled Trials and Subgroup Analysis by Sex and Diabetes Status
Source: PLoS One. 2014 Oct 31;9(10):e90286. doi: 10.1371/journal.pone.0090286 (PMC4215843; doi:10.1371/journal.pone.0090286)
Supplement: Table S3 — Details of the included studies in the subgroup analyses by sex. (DOCX) [file pone.0090286.s006.docx]

**Table S3. Number of subgroup analyses by sex.**

|  |  | **Male** | | | |  |  | **Female** | | | |
| --- | --- | --- | --- | --- | --- | --- | --- | --- | --- | --- | --- |
| **Outcomes** | **Studies** | **Aspirin** | | **Control** | |  | **Studies** | **Aspirin** | | **Control** | |
|  |  | **No. of events** | **No. of totals** | **No. of events** | **No. of totals** |  |  | **No. of events** | **No. of totals** | **No. of events** | **No. of totals** |
| **MCEs** | PHS | 307 | 11037 | 370 | 11034 |  |  |  |  |  |  |
|  | BDT | 289 | 3429 | 147 | 1710 |  |  |  |  |  |  |
|  | TPT | 228 | 2545 | 260 | 2540 |  | WHS | 477 | 19934 | 522 | 19942 |
|  | HOT | 173 | 4962 | 207 | 4945 |  | HOT | 104 | 4437 | 134 | 4446 |
|  | PPP | 28 | 949 | 38 | 963 |  | PPP | 17 | 1277 | 26 | 1306 |
|  | ETDRS | 139 | 1031 | 176 | 1065 |  | ETDRS | 120 | 825 | 133 | 790 |
|  | POPADAD | 68 | 286 | 62 | 277 |  | POPADAD | 48 | 352 | 55 | 361 |
|  | JPAD | 40 | 706 | 51 | 681 |  | JPAD | 28 | 556 | 35 | 596 |
|  | AAA | 96 | 481 | 83 | 473 |  | AAA | 85 | 1194 | 93 | 1202 |
|  |  |  |  |  |  |  |  |  |  |  |  |
| **MI** | PHS | 139 | 11037 | 239 | 11034 |  |  |  |  |  |  |
|  | BDT | 169 | 3429 | 88 | 1710 |  |  |  |  |  |  |
|  | TPT | 154 | 2545 | 190 | 2540 |  | WHS | 198 | 19934 | 193 | 19942 |
|  | HOT | 54 | 4962 | 93 | 4945 |  | HOT | 29 | 4437 | 35 | 4446 |
|  | PPP | 11 | 949 | 22 | 963 |  | PPP | 8 | 1277 | 6 | 1306 |
|  | ETDRS | 89 | 1031 | 128 | 1065 |  | ETDRS | 81 | 825 | 100 | 790 |
|  |  |  |  |  |  |  |  |  |  |  |  |
| **Stroke** | PHS | 119 | 11037 | 98 | 11034 |  |  |  |  |  |  |
|  | BDT | 91 | 3429 | 39 | 1710 |  |  |  |  |  |  |
|  | TPT | 47 | 2545 | 48 | 2540 |  | WHS | 221 | 19678 | 266 | 19942 |
|  | HOT | 94 | 4962 | 80 | 4945 |  | HOT | 54 | 4437 | 67 | 4446 |
|  | ETDRS | 45 | 1031 | 42 | 1065 |  | ETDRS | 38 | 825 | 30 | 790 |
|  | PPP | 10 | 949 | 13 | 963 |  | PPP | 6 | 1277 | 11 | 1306 |
|  |  |  |  |  |  |  |  |  |  |  |  |
| **Ischemic  stroke** | PHS | 91 | 11037 | 82 | 11034 |  |  |  |  |  |  |
|  | BDT | 21 | 3429 | 7 | 1710 |  |  |  |  |  |  |
|  | TPT | 21 | 2545 | 33 | 2567 |  | WHS | 170 | 19934 | 221 | 19942 |
|  | PPP | 8 | 949 | 7 | 963 |  | PPP | 6 | 1277 | 9 | 1306 |
|  |  |  |  |  |  |  |  |  |  |  |  |
| **Hemorrhagic  stroke** | PHS | 23 | 11037 | 12 | 11034 |  |  |  |  |  |  |
|  | BDT | 13 | 3429 | 6 | 1710 |  |  |  |  |  |  |
|  | TPT | 12 | 2545 | 6 | 2540 |  | WHS | 51 | 19934 | 41 | 19942 |
|  | PPP | 2 | 949 | 1 | 963 |  | PPP | 0 | 1277 | 2 | 1306 |
|  |  |  |  |  |  |  |  |  |  |  |  |
| **Cardiovascular  mortality** | PHS | 81 | 11037 | 83 | 11034 |  |  |  |  |  |  |
|  | BDT | 148 | 3429 | 79 | 1710 |  |  |  |  |  |  |
|  | TPT | 101 | 2545 | 81 | 2540 |  | WHS | 120 | 19934 | 126 | 19942 |
|  | HOT | 83 | 4962 | 93 | 4945 |  | HOT | 50 | 4437 | 47 | 4446 |
|  | PPP | 11 | 949 | 16 | 963 |  | PPP | 6 | 1277 | 15 | 1306 |
|  | ETDRS | 89 | 1031 | 109 | 1065 |  | ETDRS | 83 | 825 | 99 | 790 |
|  | POPADAD | 26 | 286 | 19 | 277 |  | POPADAD | 17 | 352 | 16 | 361 |
|  |  |  |  |  |  |  |  |  |  |  |  |
| **Total mortality** | PHS | 217 | 11037 | 227 | 11034 |  |  |  |  |  |  |
|  | BDT | 270 | 3429 | 151 | 1710 |  |  |  |  |  |  |
|  | TPT | 216 | 2545 | 205 | 2540 |  | WHS | 609 | 19934 | 642 | 19942 |
|  | HOT | 173 | 4962 | 207 | 4945 |  | HOT | 111 | 4437 | 99 | 4446 |
|  | PPP | 42 | 949 | 44 | 963 |  | PPP | 20 | 1277 | 34 | 1306 |
|  | ETDRS | 128 | 1031 | 147 | 1065 |  | ETDRS | 96 | 825 | 128 | 790 |
|  |  |  |  |  |  |  |  |  |  |  |  |
| **Major bleeding** | PHS | 48 | 11037 | 28 | 10979 |  |  |  |  |  |  |
|  | BDT | 29 | 3429 | 7 | 1710 |  |  |  |  |  |  |
|  | TPT | 20 | 2545 | 13 | 2540 |  | WHS | 127 | 19934 | 91 | 19942 |
|  | HOT | 83 | 4962 | 50 | 5035 |  | HOT | 47 | 4437 | 25 | 4446 |
|  | PPP | 15 | 949 | 4 | 963 |  | PPP | 9 | 1277 | 2 | 1306 |
