# Supplementary material for: Aspirin for Primary Prevention of Cardiovascular Events: Meta-Analysis of Randomized Controlled Trials and Subgroup Analysis by Sex and Diabetes Status
Source: PLoS One. 2014 Oct 31;9(10):e90286. doi: 10.1371/journal.pone.0090286 (PMC4215843; doi:10.1371/journal.pone.0090286)
Supplement: Table S4 — A. Details of the included studies in the subgroup analyses by diabetes status. B. Details of the included studies in the subgroup analyses by diabetes rate. (DOCX) [file pone.0090286.s007.docx]

**Table S4-A. Number of subgroup analyses by diabetes status.**

|  |  | **Diabetes** | | | |  |  | **Non-diabetes** | | | |
| --- | --- | --- | --- | --- | --- | --- | --- | --- | --- | --- | --- |
| **Outcomes** | **Studies** | **Aspirin** | | **Control** | |  | **Studies** | **Aspirin** | | **Control** | |
|  |  | **No. of events** | **No. of totals** | **No. of events** | **No. of totals** |  |  | **No. of events** | **No. of totals** | **No. of events** | **No. of totals** |
| **MCEs** | BDT | 13 | 69 | 6 | 32 |  | BDT | 276 | 3360 | 141 | 1678 |
|  | TPT | 4 | 29 | 6 | 39 |  | TPT | 224 | 2516 | 254 | 2501 |
|  | HOT | 47 | 752 | 54 | 749 |  | HOT | 341 | 8647 | 371 | 8642 |
|  | PPP | 20 | 519 | 22 | 512 |  | PPP | 25 | 1707 | 42 | 1757 |
|  | WHS | 58 | 538 | 62 | 499 |  | WHS | 419 | 19396 | 460 | 19443 |
|  |  |  |  |  |  |  |  |  |  |  |  |
| **MI** | PHS | 11 | 275 | 26 | 258 |  | PHS | 128 | 10762 | 213 | 10776 |
|  | HOT | 11 | 752 | 18 | 749 |  | HOT | 146 | 8647 | 166 | 8642 |
|  | PPP | 5 | 519 | 10 | 512 |  | PPP | 14 | 1707 | 18 | 1757 |
|  | WHS | 36 | 538 | 24 | 499 |  | WHS | 162 | 19396 | 169 | 19443 |
|  | AAA | 1 | 45 | 5 | 43 |  | AAA | 67 | 1630 | 65 | 1632 |
|  |  |  |  |  |  |  |  |  |  |  |  |
| **Stroke** | PHS | 16 | 275 | 10 | 258 |  | PHS | 103 | 10762 | 88 | 10776 |
|  | BDT | 3 | 69 | 1 | 32 |  | BDT | 88 | 3360 | 38 | 1678 |
|  | TPT | 1 | 29 | 2 | 39 |  | TPT | 46 | 2516 | 46 | 2501 |
|  | HOT | 20 | 752 | 22 | 749 |  | HOT | 126 | 8647 | 126 | 8642 |
|  | PPP | 9 | 519 | 10 | 512 |  | PPP | 7 | 1707 | 14 | 1757 |
|  | WHS | 15 | 538 | 31 | 499 |  | WHS | 206 | 19140 | 235 | 19443 |
|  | AAA | 0 | 45 | 2 | 43 |  | AAA | 44 | 1630 | 48 | 1632 |
|  |  |  |  |  |  |  |  |  |  |  |  |
| **Cardiovascular death** | HOT | 23 | 752 | 26 | 749 |  | HOT | 110 | 8647 | 114 | 8642 |
|  | PPP | 10 | 519 | 8 | 512 |  | PPP | 7 | 1707 | 23 | 1757 |
|  | AAA | 2 | 45 | 5 | 43 |  | AAA | 33 | 1630 | 25 | 1632 |
|  |  |  |  |  |  |  |  |  |  |  |  |
| **Total death** | HOT | 40 | 752 | 36 | 749 |  | HOT | 244 | 8647 | 269 | 8642 |
|  | PPP | 25 | 519 | 20 | 512 |  | PPP | 37 | 1707 | 58 | 1757 |
|  | AAA | 10 | 45 | 13 | 43 |  | AAA | 166 | 1630 | 173 | 1632 |

**Table S4-B. Number of subgroup analyses by diabetes rate.**

|  |  | **Diabetes rate <50%** | | | |  |  | **Diabetes rate >50%** | | | |
| --- | --- | --- | --- | --- | --- | --- | --- | --- | --- | --- | --- |
| **Outcomes** | **Studies** | **Aspirin** | | **Control** | |  | **Studies** | **Aspirin** | | **Control** | |
|  |  | **No. of events** | **No. of totals** | **No. of events** | **No. of totals** |  |  | **No. of events** | **No. of totals** | **No. of events** | **No. of totals** |
| **MCEs** | PHS | 307 | 11037 | 370 | 11034 |  | POPADAD | 119 | 638 | 123 | 638 |
|  | BDT | 289 | 3429 | 147 | 1710 |  | JPAD | 40 | 1262 | 46 | 1277 |
|  | TPT | 228 | 2545 | 260 | 2540 |  | ETDRS | 350 | 1856 | 379 | 1855 |
|  | HOT | 388 | 9399 | 425 | 9391 |  | CLIPS | 7 | 185 | 20 | 181 |
|  | PPP | 45 | 2226 | 64 | 2269 |  |  |  |  |  |  |
|  | WHS | 477 | 19934 | 522 | 19942 |  |  |  |  |  |  |
|  | AAA | 134 | 1675 | 136 | 1675 |  |  |  |  |  |  |
|  | APLASA | 3 | 48 | 0 | 50 |  |  |  |  |  |  |
|  | ECLAP | 5 | 253 | 13 | 265 |  |  |  |  |  |  |
|  |  |  |  |  |  |  |  |  |  |  |  |
| **MI** | PHS | 139 | 11037 | 239 | 11034 |  | POPADAD | 90 | 638 | 82 | 638 |
|  | BDT | 169 | 3429 | 88 | 1710 |  | JPAD | 12 | 1262 | 14 | 1277 |
|  | TPT | 154 | 2545 | 190 | 2540 |  | ETDRS | 241 | 1856 | 283 | 1855 |
|  | HOT | 157 | 9399 | 184 | 9391 |  | CLIPS | 2 | 185 | 11 | 181 |
|  | PPP | 19 | 2226 | 28 | 2269 |  |  |  |  |  |  |
|  | WHS | 198 | 19934 | 193 | 19942 |  |  |  |  |  |  |
|  | AAA | 68 | 1675 | 70 | 1675 |  |  |  |  |  |  |
|  | APLASA | 1 | 48 | 0 | 50 |  |  |  |  |  |  |
|  | ECLAP | 1 | 253 | 2 | 265 |  |  |  |  |  |  |
|  | ACBS | 7 | 188 | 4 | 184 |  |  |  |  |  |  |
|  |  |  |  |  |  |  |  |  |  |  |  |
| **Stroke** | PHS | 119 | 11037 | 98 | 11034 |  | POPADAD | 37 | 638 | 50 | 638 |
|  | BDT | 91 | 3429 | 39 | 1710 |  | JPAD | 28 | 1262 | 32 | 1277 |
|  | TPT | 47 | 2545 | 48 | 2540 |  | ETDRS | 92 | 1856 | 78 | 1855 |
|  | HOT | 146 | 9399 | 148 | 9391 |  | CLIPS | 4 | 185 | 7 | 181 |
|  | PPP | 16 | 2226 | 24 | 2269 |  |  |  |  |  |  |
|  | WHS | 221 | 19678 | 266 | 19942 |  |  |  |  |  |  |
|  | AAA | 44 | 1675 | 50 | 1675 |  |  |  |  |  |  |
|  | ECLAP | 0 | 253 | 5 | 265 |  |  |  |  |  |  |
|  | ACBS | 11 | 188 | 10 | 184 |  |  |  |  |  |  |
|  |  |  |  |  |  |  |  |  |  |  |  |
| **Ischemic** | PHS | 91 | 11037 | 82 | 11034 |  | POPADAD | 5 | 638 | 3 | 638 |
| **stroke** | BDT | 21 | 3429 | 7 | 1710 |  | JPAD | 22 | 1262 | 24 | 1277 |
|  | TPT | 21 | 2545 | 33 | 2540 |  |  |  |  |  |  |
|  | PPP | 14 | 2226 | 16 | 2269 |  |  |  |  |  |  |
|  | WHS | 170 | 19934 | 221 | 19942 |  |  |  |  |  |  |
|  | AAA | 30 | 1675 | 37 | 1675 |  |  |  |  |  |  |
|  | ECLAP | 0 | 253 | 4 | 265 |  |  |  |  |  |  |
|  |  |  |  |  |  |  |  |  |  |  |  |
| **Hemorrhagic** | PHS | 23 | 11037 | 12 | 11034 |  | POPADAD | 2 | 638 | 3 | 638 |
| **stroke** | BDT | 13 | 3429 | 6 | 1710 |  | JPAD | 5 | 1262 | 3 | 1277 |
|  | TPT | 12 | 2545 | 6 | 2540 |  |  |  |  |  |  |
|  | PPP | 2 | 2226 | 3 | 2269 |  |  |  |  |  |  |
|  | WHS | 51 | 19934 | 41 | 19942 |  |  |  |  |  |  |
|  | AAA | 5 | 1675 | 4 | 1675 |  |  |  |  |  |  |
|  | ECLAP | 0 | 253 | 1 | 265 |  |  |  |  |  |  |
|  |  |  |  |  |  |  |  |  |  |  |  |
| **Cardiovascular death** | PHS | 81 | 11037 | 38 | 11034 |  | POPADAD | 35 | 638 | 26 | 638 |
|  | BDT | 148 | 3429 | 79 | 1710 |  | JPAD | 1 | 1262 | 10 | 1277 |
|  | TPT | 101 | 2545 | 81 | 2540 |  | ETDRS | 244 | 1856 | 275 | 1855 |
|  | HOT | 133 | 9399 | 140 | 9391 |  | CLIPS | 5 | 185 | 4 | 181 |
|  | PPP | 17 | 2226 | 31 | 2269 |  |  |  |  |  |  |
|  | WHS | 120 | 19934 | 126 | 19942 |  |  |  |  |  |  |
|  | AAA | 35 | 1675 | 30 | 1675 |  |  |  |  |  |  |
|  | ECLAP | 3 | 253 | 8 | 265 |  |  |  |  |  |  |
|  | ACBS | 10 | 188 | 7 | 184 |  |  |  |  |  |  |
|  |  |  |  |  |  |  |  |  |  |  |  |
| **Total death** | PHS | 217 | 11037 | 227 | 11034 |  | POPADAD | 94 | 638 | 101 | 638 |
|  | BDT | 270 | 3429 | 151 | 1710 |  | JPAD | 34 | 1262 | 38 | 1277 |
|  | TPT | 216 | 2545 | 205 | 2540 |  | ETDRS | 340 | 1856 | 366 | 1855 |
|  | HOT | 284 | 9399 | 305 | 9391 |  | CLIPS | 7 | 185 | 4 | 181 |
|  | PPP | 62 | 2226 | 78 | 2269 |  |  |  |  |  |  |
|  | WHS | 609 | 19934 | 642 | 19942 |  |  |  |  |  |  |
|  | AAA | 176 | 1675 | 186 | 1675 |  |  |  |  |  |  |
|  | ECLAP | 9 | 253 | 18 | 265 |  |  |  |  |  |  |
|  | ACBS | 11 | 188 | 13 | 184 |  |  |  |  |  |  |
|  |  |  |  |  |  |  |  |  |  |  |  |
| **Major bleeding** | PHS | 48 | 11037 | 28 | 10979 |  | POPADAD | 28 | 638 | 31 | 638 |
|  | BDT | 29 | 3429 | 7 | 1710 |  | JPAD | 12 | 1262 | 4 | 1277 |
|  | TPT | 20 | 2545 | 13 | 2540 |  | ETDRS | 37 | 1856 | 37 | 1855 |
|  | HOT | 136 | 9399 | 78 | 9391 |  | CLIPS | 4 | 185 | 0 | 181 |
|  | PPP | 24 | 2226 | 6 | 2269 |  |  |  |  |  |  |
|  | WHS | 127 | 19934 | 91 | 19942 |  |  |  |  |  |  |
|  | AAA | 34 | 1675 | 20 | 1675 |  |  |  |  |  |  |
|  | ECLAP | 23 | 253 | 14 | 265 |  |  |  |  |  |  |
