# Supplementary material for: Aspirin for Primary Prevention of Cardiovascular Events: Meta-Analysis of Randomized Controlled Trials and Subgroup Analysis by Sex and Diabetes Status
Source: PLoS One. 2014 Oct 31;9(10):e90286. doi: 10.1371/journal.pone.0090286 (PMC4215843; doi:10.1371/journal.pone.0090286)
Supplement: Text S1 — Study protocol. (DOC) [file pone.0090286.s009.doc]

**Text S1. Study protocol.**

First drafted in November 2012

Updated in April 2013

**Aspirin for primary prevention of cardiovascular events: meta-analysis of randomized controlled trials** **and subgroup analysis by sex and diabetes status**

Manling Xie, Zhilei Shan, Liegang Liu

Department of Nutrition and Food Hygiene, Hubei Key Laboratory of Food Nutrition and Safety, School of Public Health, Tongji Medical College, Huazhong University of Science & Technology

**Objectives and aims:**

To evaluate the benefits and harms of aspirin for the primary prevention of CVD and determine whether the effects vary by sex and diabetes status.

**Background：**

The burden of cardiovascular disease (CVD) is substantial. The most recent (2013) statistics on heart disease and stroke from the American Heart Association (AHA) estimate that the annual direct and indirect cost of CVD and stroke in the United States alone are $523 billion . From 2000 to 2010, the total number of inpatient cardiovascular operations and procedures increased 28%, from 5,939,000 to 7,588,000. By 2030, 40.8% of the US population is projected to have some form of CVD, and the annual cost will increase to $1.13 trillion. These strong upward trends underline the importance of primary prevention for those who are already at high risk of CVD.

The use of low-dose aspirin for primary prevention of CVD is recommended by many key guidelines . However, a recent published study and a review stated that the benefit of aspirin for the primary prevention of cardiovascular events was relatively small for individuals regardless of diabetic status and could easily be offset by the risk of hemorrhage. These studies challenge current recommendations, which are based on outcomes from several meta-analyses , prompting re-evaluation of the efficacy of aspirin. An important sex-specific meta-analysis showed that the effects of aspirin varied by sex . However, it was conducted in 2006 and included only six primary prevention trials. In addition, the results were not confirmed in the Antithrombotic Trialists’ Collaboration meta-analysis and a recent publication which did not find significant sex different in treatment effect . Several guidelines recommend aspirin for the primary prevention of cardiovascular events in patients with diabetes at risk of CVD, but others do not. This conflict reflects the lack of definitive evidence. Existing recommendations are primarily based on extrapolations from indirect evidence, given the absence of statistically significant results in published meta-analyses in diabetics .

Therefore, we performed a new meta-analysis to re-assess the effects of aspirin for primary prevention of CVD and to investigate whether the effects vary by sex and diabetes status. Compared to the previous sex-specific meta-analyses, we enrolled almost twice that of previously published data. Given the limited power to detect interactions, even in a meta-analysis combining the results from several studies, we used multiple statistical methods to examine the diabetes-aspirin interaction and sex-aspirin interaction and their consistent results strengthen our conclusions.

**Research Plan:**

**A) Methods of the review**

The study will be conducted according to the PRISMA statement for the conduct of meta-analyses.

**B) Data sources and searches**

We identified trials by searching Medline, Embase, and Central (the Cochrane Central Register of Controlled Trials) from inception to December 2012. Reference lists from previously published relevant systematic reviews were also screened for additional studies and guidelines of aspirin use for primary prevention of CVD will be searched as well.

**Details of the search strategies**

First we searched terms “aspirin*” [MeSH] and term “primary prevention”. Then the Boolean term “AND” was used to combine these two terms. Highly sensitive filters were used to limit results to randomized controlled trials and human studies. We searched only studies published in English. A similar search strategy was used for Embase and Central.

**C) Study selection:**

**Types of studies:**

Randomized controlled trials (RCTs) comparing the effect of aspirin with placebo or control in people without pre-existing CVD on outcomes of interest were eligible for inclusion**.**

**Types of participants:**

Selection criteria included:

1) Prospective, randomized, controlled, open, or blinded trials.

2) Participants without clinical CVD (e.g., established or symptomatic) were randomly assigned to aspirin (any dose) versus placebo or control group for the primary prevention of CVD.

3) Trials carried out on a background of anticoagulation were eligible.

4) Follow-up had to exceed 90 days. We excluded studies with fewer than 90 days of follow-up, because such short follow-up would not permit detection of cardiovascular outcomes related to aspirin treatment for primary prevention.

**Type of intervention:**

For primary prevention of CVD

**Type of outcome measures*:***

1. major cardiovascular events (MCE, defined as death from cardiovascular causes, nonfatal myocardial infarction, and nonfatal stroke)Cardiovascular mortality
2. myocardial infarction (MI, fatal and nonfatal)
3. stroke (fatal and nonfatal)
4. ischemic stroke
5. hemorrhagic stroke
6. cardiovascular mortality
7. total mortality (death from any cause)
8. major bleeding

Definitions for major bleeding varied across studies, and participant-level data were unavailable to allow reclassification according to standard criteria . Among all bleeding events, the gastrointestinal hemorrhage is one of the most common and serious complications of long-term aspirin use.

**Ongoing Trials :**

1). the Aspirin to Reduce Risk of Initial Vascular Events (ARRIVE) Study (<http://www.arrive-study.com/EN/study.cfm>)

2). Aspirin in Reducing Events in the Elderly (ASPREE) .

3). A Study of Cardiovascular Events in Diabetes (ASCEND, International Standard Randomised Controlled Trial Number ISRCTN60635500, <http://www.ctsu.ox.ac.uk/ascend/>)

4). the Aspirin and Simvastatin Combination for Cardiovascular Events Prevention Trial in Diabetes (ACCEPT-D, Current Controlled Trials ISRCTN48110081) , which enrolled more than 15,000 diabetic patients without prior cardiovascular events to assess the effect of aspirin in the prevention of cardiovascular events.

These trials may provide sufficient data to identify patients who derive the most benefit from aspirin therapy.

**References**

1. Go AS, Mozaffarian D, Roger VL, Benjamin EJ, Berry JD, et al. (2013) Heart disease and stroke statistics--2013 update: a report from the American Heart Association. Circulation 127: e6-e245.

2. (2009) Aspirin for the prevention of cardiovascular disease: U.S. Preventive Services Task Force recommendation statement. Ann Intern Med 150: 396-404.

3. Wolff T, Miller T, Ko S (2009) Aspirin for the primary prevention of cardiovascular events: an update of the evidence for the U.S. Preventive Services Task Force. Ann Intern Med 150: 405-410.

4. De Berardis G, Lucisano G, D'Ettorre A, Pellegrini F, Lepore V, et al. (2012) Association of aspirin use with major bleeding in patients with and without diabetes. JAMA 307: 2286-2294.

5. Kappagoda T, Amsterdam E (2012) Aspirin for primary prevention of myocardial infarction: what is the evidence? J Cardiopulm Rehabil Prev 32: 1-8.

6. Berger JS, Roncaglioni MC, Avanzini F, Pangrazzi I, Tognoni G, et al. (2006) Aspirin for the primary prevention of cardiovascular events in women and men: a sex-specific meta-analysis of randomized controlled trials. JAMA 295: 306-313.

7. Baigent C, Blackwell L, Collins R, Emberson J, Godwin J, et al. (2009) Aspirin in the primary and secondary prevention of vascular disease: collaborative meta-analysis of individual participant data from randomised trials. Lancet 373: 1849-1860.

8. Berger JS, Lala A, Krantz MJ, Baker GS, Hiatt WR (2011) Aspirin for the prevention of cardiovascular events in patients without clinical cardiovascular disease: a meta-analysis of randomized trials. Am Heart J 162: 115-124 e112.

9. Rothwell PM, Price JF, Fowkes FG, Zanchetti A, Roncaglioni MC, et al. (2012) Short-term effects of daily aspirin on cancer incidence, mortality, and non-vascular death: analysis of the time course of risks and benefits in 51 randomised controlled trials. Lancet 379: 1602-1612.

10. ., Ghahramani P, Jackson PR, Wallis EJ, Ramsay LE (2001) Aspirin for primary prevention of coronary heart disease: safety and absolute benefit related to coronary risk derived from meta-analysis of randomised trials. Heart 85: 265-271.

11. Seshasai SR, Wijesuriya S, Sivakumaran R, Nethercott S, Erqou S, et al. (2012) Effect of aspirin on vascular and nonvascular outcomes: meta-analysis of randomized controlled trials. Arch Intern Med 172: 209-216.

12. Buse JB, Ginsberg HN, Bakris GL, Clark NG, Costa F, et al. (2007) Primary prevention of cardiovascular diseases in people with diabetes mellitus: a scientific statement from the American Heart Association and the American Diabetes Association. Circulation 115: 114-126.

13. (2008) Type 2 Diabetes: National Clinical Guideline for Management in Primary and Secondary Care (Update). London: Royal College of Physicians of London.

14. Ryden L, Standl E, Bartnik M, Van den Berghe G, Betteridge J, et al. (2007) Guidelines on diabetes, pre-diabetes, and cardiovascular diseases: executive summary. The Task Force on Diabetes and Cardiovascular Diseases of the European Society of Cardiology (ESC) and of the European Association for the Study of Diabetes (EASD). Eur Heart J 28: 88-136.

15. De Berardis G, Sacco M, Strippoli GFM, Pellegrini F, Graziano G, et al. (2009) Aspirin for primary prevention of cardiovascular events in people with diabetes: meta-analysis of randomised controlled trials. Bmj 339: b4531-b4531.

16. Calvin AD, Aggarwal NR, Murad MH, Shi Q, Elamin MB, et al. (2009) Aspirin for the primary prevention of cardiovascular events: a systematic review and meta-analysis comparing patients with and without diabetes. Diabetes Care 32: 2300-2306.

17. Younis N, Williams S, Ammori B, Soran H (2010) Role of aspirin in the primary prevention of cardiovascular disease in diabetes mellitus: a meta-analysis. Expert Opin Pharmacother 11: 1459-1466.

18. Zhang C, Sun A, Zhang P, Wu C, Zhang S, et al. (2010) Aspirin for primary prevention of cardiovascular events in patients with diabetes: A meta-analysis. Diabetes Res Clin Pract 87: 211-218.

19. Pignone M, Alberts MJ, Colwell JA, Cushman M, Inzucchi SE, et al. (2010) Aspirin for primary prevention of cardiovascular events in people with diabetes: a position statement of the American Diabetes Association, a scientific statement of the American Heart Association, and an expert consensus document of the American College of Cardiology Foundation. Diabetes Care 33: 1395-1402.

20. Stavrakis S, Stoner JA, Azar M, Wayangankar S, Thadani U (2011) Low-dose aspirin for primary prevention of cardiovascular events in patients with diabetes: a meta-analysis. Am J Med Sci 341: 1-9.

21. Butalia S, Leung AA, Ghali WA, Rabi DM (2011) Aspirin effect on the incidence of major adverse cardiovascular events in patients with diabetes mellitus: a systematic review and meta-analysis. Cardiovasc Diabetol 10: 25.

22. Simpson SH, Gamble JM, Mereu L, Chambers T (2011) Effect of aspirin dose on mortality and cardiovascular events in people with diabetes: a meta-analysis. J Gen Intern Med 26: 1336-1344.

23. Schnell O, Erbach M, Hummel M (2012) Primary and secondary prevention of cardiovascular disease in diabetes with aspirin. Diab Vasc Dis Res 9: 245-255.

24. Altman DG, Bland JM (2003) Interaction revisited: the difference between two estimates. BMJ 326: 219.

25. Chesebro JH, Knatterud G, Roberts R, Borer J, Cohen LS, et al. (1987) Thrombolysis in Myocardial Infarction (TIMI) Trial, Phase I: A comparison between intravenous tissue plasminogen activator and intravenous streptokinase. Clinical findings through hospital discharge. Circulation 76: 142-154.

26. (1993) An international randomized trial comparing four thrombolytic strategies for acute myocardial infarction. The GUSTO investigators. N Engl J Med 329: 673-682.

27. Reid CM, Storey E, Wong TY, Woods R, Tonkin A, et al. (2012) Aspirin for the prevention of cognitive decline in the elderly: rationale and design of a neuro-vascular imaging study (ENVIS-ion). BMC Neurol 12: 3.

28. De Berardis G, Sacco M, Evangelista V, Filippi A, Giorda CB, et al. (2007) Aspirin and Simvastatin Combination for Cardiovascular Events Prevention Trial in Diabetes (ACCEPT-D): design of a randomized study of the efficacy of low-dose aspirin in the prevention of cardiovascular events in subjects with diabetes mellitus treated with statins. Trials 8: 21.
